# Review on Dog Rabies Vaccination Coverage in Africa: A Question of Dog Accessibility or Cost Recovery?

**DOI:** 10.1371/journal.pntd.0003447

**Published:** 2015-02-03

**Authors:** Tariku Jibat, Henk Hogeveen, Monique C. M. Mourits

**Affiliations:** 1 Business Economics Group, Wageningen University, Wageningen, The Netherlands; 2 College of Veterinary Medicine and Agriculture, Addis Ababa University, Debre Zeit, Ethiopia; The Global Alliance for Rabies Control, UNITED STATES

## Abstract

**Background:**

Rabies still poses a significant human health problem throughout most of Africa, where the majority of the human cases results from dog bites. Mass dog vaccination is considered to be the most effective method to prevent rabies in humans. Our objective was to systematically review research articles on dog rabies parenteral vaccination coverage in Africa in relation to dog accessibility and vaccination cost recovery arrangement (i.e.free of charge or owner charged).

**Methodology/Principal Findings:**

A systematic literature search was made in the databases of CAB abstracts (EBSCOhost and OvidSP), Scopus, Web of Science, PubMed, Medline (EBSCOhost and OvidSP) and AJOL (African Journal Online) for peer reviewed articles on 1) rabies control, 2) dog rabies vaccination coverage and 3) dog demography in Africa. Identified articles were subsequently screened and selected using predefined selection criteria like year of publication (viz. ≥ 1990), type of study (cross sectional), objective(s) of the study (i.e. vaccination coverage rates, dog demographics and financial arrangements of vaccination costs), language of publication (English) and geographical focus (Africa). The selection process resulted in sixteen peer reviewed articles which were used to review dog demography and dog ownership status, and dog rabies vaccination coverage throughout Africa. The main review findings indicate that 1) the majority (up to 98.1%) of dogs in African countries are owned (and as such accessible), 2) puppies younger than 3 months of age constitute a considerable proportion (up to 30%) of the dog population and 3) male dogs are dominating in numbers (up to 3.6 times the female dog population). Dog rabies parenteral vaccination coverage was compared between “free of charge” and “owner charged” vaccination schemes by the technique of Meta-analysis. Results indicate that the rabies vaccination coverage following a free of charge vaccination scheme (68%) is closer to the World Health Organization recommended coverage rate (70%) than the achieved coverage rate in owner-charged dog rabies vaccination schemes (18%).

**Conclusions/Significance:**

Most dogs in Africa are owned and accessible for parenteral vaccination against rabies if the campaign is performed “free of charge”.

## Author Summary

Rabies is one of the most fatal diseases in both humans and animals. A bite by a rabid dog is the main cause of human rabies in Africa. Parenteral mass dog vaccination is the most cost-effective tool to prevent rabies in humans. Our main objective was to review research articles on the parenteral dog rabies vaccination coverage in Africa. We aimed to review published research articles on percentage of dogs owned and percentage of dogs vaccinated against rabies, and on the relation between vaccination coverage and cost recovery. We followed the standard procedures of a systematic literature review resulting in a final review of 16 scientific articles. Our review results indicate that only a small percentage of African dogs is ownerless. Puppies younger than 3 months of age constitute a considerable proportion of the African dog population. There are considerably more male dogs than female dogs present within the dog population. The dog rabies parenteral vaccination coverage following a “free of charge” vaccination scheme (68%) is closer to World Health Organization recommended threshold coverage rate (70%) compared to the coverage rate achieved in “owner-charged” dog rabies vaccination schemes (18%). In conclusion, most dogs in Africa are owned and accessible for vaccination once the necessary financial arrangements have been made.

## Introduction

Rabies is one of the infectious diseases with the highest human case fatality rate (almost 100%)[1]. Globally, rabies is responsible for more than 60,000 human deaths, while approximately 15 million people receive rabies post exposure prophylaxis (PEP) annually. More than 95% of the global deaths occur in Asia and Africa, where canine rabies is enzootic [2]. Africa contributes to 43% of the human deaths due to rabies [3]. In addition to human life losses, rabies is also a cause of substantial livestock losses [4] and a threat to rare carnivores like the Ethiopian wolf (*Canis simensis*) [5] and the African wild dog (*Lycaon pictus*) [6]. Despite these consequences, rabies has been seriously neglected in Africa [7].

The main cause of transmission of rabies to human in Africa is by a bite of a rabid dog [8]. Once bitten by a rabid dog, development of the disease in human can be prevented by an appropriate post-exposure prophylaxis (PEP). However, PEP is relatively expensive and not always available. Moreover, PEP lacks long-term benefits as it will not stop the virus transmission from rabid dogs to other humans or dogs [9]. Dog rabies parenteral vaccination is therefore more cost-effective measure in preventing human rabies [10].

To eliminate rabies from the dog population in an endemic area at least 70% of the dog population needs to be vaccinated during an annual rabies mass vaccination campaign [11]. In many African countries, the proportion of dogs vaccinated against rabies is far below 70% [12]. Accessibility of free roaming dogs for vaccination is often mentioned as an operational constraint [13] with the assumption that parenteral dog vaccination requires catching and restraining dogs physically. Catching free roaming dogs is easier if the dogs are owned. Therefore, dog ownership is an important factor in determining the percentage of dogs vaccinated during a campaign. Dog ownership status and management factors in developing countries in relation to dog rabies vaccination have extensively been addressed in literature (see for example [14]). But, as developing countries at different continents have a wide variation in social and cultural context, studies on African specific socio-economic situations related to dogs and rabies are a necessity for a valid interpretation and practical application of effective vaccination campaigns in Africa. Besides, there is no valid evidence to what extent charging owners for the costs of dog vaccination against rabies contribute to a low vaccination coverage.

Therefore, the objective of our study is to systematically review articles on parenteral vaccination coverage on dog rabies achieved in Africa, in relation to dog demographics and financial arrangements on vaccination costs.

## Methods

### Article search strategy

To obtain insight in the trend of peer reviewed articles focussing on “dog rabies control in Africa” during the last 20 years (1994–2013), a systematic search was made in the databases of CAB abstracts (EBSCOhost and OvidSP), Scopus, Web of Science, PubMed, Medline (EBSCOhost and OvidSP) and AJOL (African Journal Online).

Subsequently, a search was made in the above mentioned databases for peer reviewed articles in the themes: 1) “rabies control”, 2) “dog vaccination coverage” and 3) “dog demography”. All theme searches were limited to papers regarding the continent of Africa. The search for each theme was conducted in the search items “title/abstract/key words” using the following search protocol: 1) ((dog? OR canine OR livestock OR human? OR wild? life) AND rabies AND control AND Africa?), 2) ((dog? OR canine) AND rabies AND vaccine* AND coverage AND Africa?) and 3) ((dog OR canine) AND (demography OR population) AND Africa?). The search protocol was designed, based on standard procedures of a systematic literature search [15]. However, as the AJOL database webpage has no feature to select the search protocol in title/abstract/keywords, the search in AJOL was done within the entire article. The systematic literature search included articles published between 1990 and January 2014.

### Framework for screening and selection criteria

**Figure 1:**
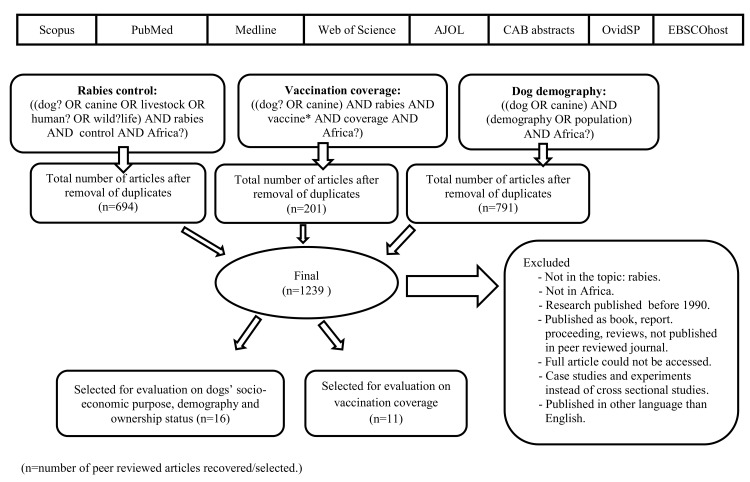
Systematic literature review framework (n = number of peer reviewed articles recovered/selected).

Publications were screened systematically according to the schematic framework as shown in Fig. 1 using EndNote X5 (Endnote @ 2013) reference manager. First, an evaluation of titles and abstracts was performed followed by a removal of duplicates (i.e., publications indexed in more than one databases and published in more than one format, including conference proceedings and book chapters). Several inclusion and exclusion criteria were considered including year of publication (viz. ≥ 1990), type of study (cross sectional), objective(s) of the study (parenteral vaccination coverage rates on dog rabies in Africa, dog demographics and financial arrangements with respect to vaccination costs), language of publication (English) and geographical focus (Africa). As a result, a publication could be excluded for more than one reason, making it impractical to reflect the number of publications excluded per criteria. Articles, of which the full text was not electronically available, were requested from the Royal College of Veterinary Surgeons Trust Library (in United Kingdom).

For each selected article, a record was made in Microsoft Excel describing the studied dog population by the main purpose for keeping dogs, dog demography (mean age, age distribution, sex ratio age and sex distribution), ownership status (percentage of owned, free roaming and ownerless dogs), and parenteral vaccination coverage based on either”free of charge” or”owner charged” financial arrangements. The selected articles encompassed research from Southern (South Africa, Madagascar, Zimbabwe, Zambia), Central (Chad), Northern (Tunisia), Eastern (Tanzania, Kenya, Ethiopia) and Western (Nigeria) Africa. The methodological quality of candidate peer review articles was critically assessed by the Assessment of Multiple Systematic Reviews (AMSTAR) measurement tool [16, 17] and (supporting information: S1 Checklist).

### Statistical analysis

Results on dog demography (i.e. sex ratio and mean age of dogs) and ownership status were extracted from the selected articles and presented in tabular form without further analysis. In a meta-analysis, we evaluated the difference in the percentage of dogs vaccinated against rabies (i.e. vaccination coverage) by the applied financial arrangement on vaccination costs; i.e. whether the vaccination was provided for free to the dog owners or not (i.e. free versus charged). From the selected articles the presented parenteral vaccination coverage was entered as an event rate in the meta-analysis software (Comprehensive meta-analysis V2, 2013). A Forest plot was created to serve as a visual representation of the data in a combined point estimate for the free and charged vaccination study groups, bounded by its confidence interval. Statistical differences, called heterogeneity tests, between the two groups of studies were tested as indicated by I^2^ and tau square. I^2^ represents the percentage of the total variation across studies due to heterogeneity across studies within a group and across a group. It takes values from 0% to 100%, with the value of 0% indicating no observed heterogeneity. Tau square is an estimate of the between study and between group variance. If greater than 1, it suggests the presence of substantial statistical heterogeneity in each group, which is a statistical variation due to heterogeneity rather than chance between the free-of-charge and charged study groups [18, 19].

## Results

### Literature search study

**Figure 2:**
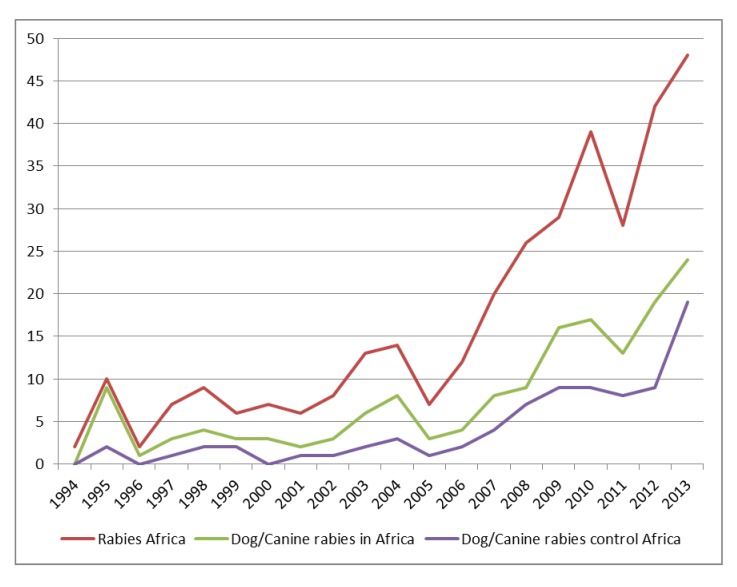
Trend in number of scientific publications on “Rabies in Africa”, “Dog/Canine rabies in Africa” and “Control of “Dog/Canine rabies in Africa” as indexed by Web of Science/Knowledge during the last 20 years.

Based on the systematic literature search using the phrase “Rabies control in Africa”, the highest number of research publications was found indexed by the Web of Science/Knowledge database. In Fig. 2 the trend in the number of scientific publications on the topics “Rabies in Africa”, “Dog/Canine rabies in Africa” and “Control of “Dog/Canine rabies in Africa” during the last 20 years indicates an increase in scientific interest on rabies in Africa (Fig. 2). While a worldwide search on “Rabies” during the same period resulted in 9,836 selected entries, only 328 of them were specifically referring to the African situation. Of these 328 papers, approximately half focussed specifically on dog rabies (“Dog/Canine rabies in Africa”, n = 172) and one fifth on dog rabies control (“Control of “Dog/Canine rabies in Africa”, n = 76).

The systematic search from the databases by the defined framework resulted in 1,239 articles (Fig. 1). After removal of all duplicates and exclusion of publications not fulfilling the selection criteria, 16 peer reviewed articles remained to be included in the study on dog demography (sex ratio and mean age of dogs) and ownership status (owned confined, owned but free roaming and proven to be ownerless). Seven of these papers have been published during the last five years. For the comparison of dog parenteral vaccination coverage against rabies related to the dog owners’ costs of vaccination, 11 peer reviewed articles with 15 entries (including four studies comparing free and owner charged vaccination arrangements) remained. The majority of these papers (7 out of 11) has been recently published (e.g., between 2009–2013).

### Socio-economic purpose, demography and ownership status of dogs

The majority (up to 98%) of dogs in African countries is kept for socio-economic purposes including guarding livestock from predators, homestead from intruders, crops from wildlife and hunting. Dogs are also used as pets, income generation means and as a protein source (Table 1) [20, 21, 22, 23]. Furthermore, puppies younger than 3 months of age constitute up to 30% of the dog population [24]. Male dogs dominate the female dogs up to 3.6 times in number within the population [22]. The mean age of the dogs varies between 1.8 and 3.4 years. Studies accounting for ownership of dogs (Table 1) showed that the percentage of ownerless dogs ranges between 0.7% and 20% of a dog population within the 11 represented African countries. Except for a study in Tanzania [24], all studies reported that more than two third of the free roaming dogs has a responsible owner[20, 22, 23, 25, 26, 27]. Owned dogs with confined housing constitute 18.5% to 60.9% of the dog population.

**Table 1:** Demography and ownership status of African dogs by the 16 selected peer reviewed papers.

Study name	Country	Purpose of dogs	Age distribution	Male: female ratio	Mean age (yr)	Owned confined (%)	Owned–free roaming (%)	Proven ownerles (%)
Cleaveland et al., 2003 [12]	Tanzania	N/A	<3 mo (12.6%), 3–6 mo (10.3%), >6 mo (77.1%)	N/A	N/A	N/A	N/A	N/A
Ratsitorahina et al., 2009 [20]	Madagascar	81.1% (security)	<6 mo (15%), 6 mo-1 yr (23.8%), >1 yr (61.2%)	1.58	N/A	18.5	70	11.5
Knobel et al., 2008 [21]	Tanzania	98.1% (security)	N/A	N/A	N/A	N/A	N/A	N/A
Yimer et al., 2012 [22]	Ethiopia	90.7% (security), 9.1% (pet)	N/A	3.63	N/A	19.1	80.9	N/A
Aiyedun et al., 2012 [23]	Nigeria	49.4% (security), 16.3% (sales), 14.6% (pet), 8.2% (hunting),3.7% (protein source)	N/A	N/A	N/A	26.3	73.7	N/A
Gsell et al., 2012 [24]	Tanzania	N/A	<3 mo (30.3%), 3–12 mo (21.7%), >12 mo (47.9%)	1.4	2.23	60.9	38.3	0.7
Van Sittert et al.,2010 [25]	South Africa	23% (security)	<3 mo (3%), 3–12 mo (18%), 1–3 yr (43%), 3–10 yr (35%), >10 yr(1%)	1.7	N/A	22	75	3
Kitala et al., 2001 [26]	Kenya	N/A	<12 mo (50.2%), 1–2 yr (17.7%), 2–3 yr (15.2%), 3–4 yr (8%), >4 yr (9%)	1.5	1.9	N/A	69	N/A
Kaare et al., 2009 [27]	Tanzania	N/A	N/A	N/A	N/A	N/A	82.3	4
Durr et al., 2009 [41]	Chad	N/A	N/A	3.4	3.4	N/A	N/A	20
Touihri et al., 2011 [50]	Tunisia	N/A	<3 mo (13%), > 3 mo (87%)	1.52	N/A	N/A	N/A	3
Kayali et al., 2003 [51]	Chad	N/A	N/A	N/A	N/A	48	N/A	7.6
Kitala et al., 1993 [52]	Kenya	99.4% (security), 0.3% (hunting and herding)	<3 mo(26%), 3–9 mo (20%), >9 mo (53%)	1.4	1.8	19.4	69	N/A
Brooks et al., 1990 [53]	Zimbabwe	N/A	N/A	1.3	2.3	N/A	N/A	N/A
De Balogh et al., 1993 [54]	Zambia	N/A	<3 mo (34%)	1.01	2	19	81	N/A
Rautenbach et al., 1991[55]	South Africa	N/A	N/A	1.29	2.6	N/A	N/A	N/A

mo = month, yr = year, N/A = not available

### Coverage rate by free versus charged parenteral mass dog vaccination schemes

**Figure 3:**
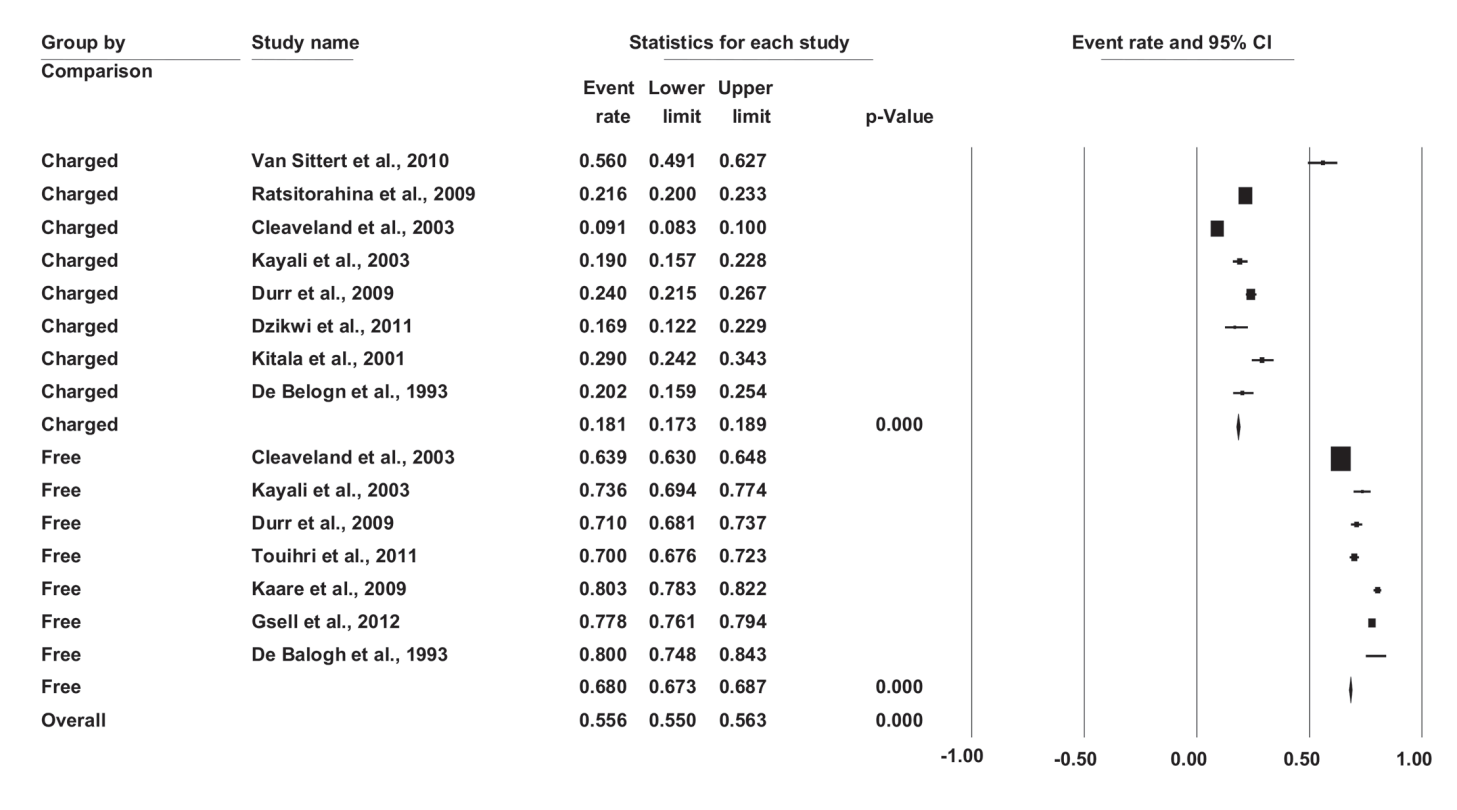
Forest plot comparing dog rabies vaccination coverage by financial arrangement.

The published studies selected for vaccination coverage comparison by vaccination costs arrangement schemes consisted of eleven studies in eight different countries representing all regions of Africa (Table 2). Four studies compared vaccination coverage under “free of charge” and “charged” arrangements schemes, four studies evaluated vaccination coverage resulting from “charged” vaccination arrangement schemes only and three studies estimated parenteral vaccination coverage by a “free-of-charge” scheme. The Forest plot (Fig. 3) shows a coverage of less than 50% in the charged groups except for one study, while all studies under free of charge arrangements resulted in a coverage above 50%. The vaccination coverage in studies based on free of charge vaccination (68%) is significant higher ((P<0.001) than the studies based on a charged vaccination campaign (18.1%).

**Table 2:** Dog rabies parenteral vaccination coverage by financial arrangement scheme (free or charged) as reflected by the 11 selected publications.

Study name	Country	Objective of the study	Vaccination coverage (%)	Financial arrangement
Van Sittert *et al*., 2010 [25]	South Africa	Dog ecology, vaccination coverage and rabies neutralising antibody levels	56	Charged
Ratsitorahina *et al*., 2009 [20]	Madagascar	Dog ecology	22	Charged
Cleaveland *et al*., 2003 [12]	Tanzania	Effect of vaccination	9	Charged
Kayali *et al*, 2003 [51]	Chad	Estimate the vaccination coverage	19	Charged
Durr *et al*., 2009 [41]	Chad	Vaccination coverage	24	Charged
Dzikwi *et al*., 2011 [44]	Nigeria	Rabies Vaccination and Immune Status	17	Charged
Kitala *et al*., 2001 [26]	Kenya	Dog ecology and demography	29	Charged
De Balogh *et al*., 1993 [54]	Zambia	Dog populations and accessibility for rabies vaccination	20	Charged
De Balogh et al., 1993 [54]	Zambia	Dog populations and accessibility for rabies vaccination	80	Free
Touihri *et al*., 2011 [50]	Tunisia	Vaccination coverage	70	Free
Cleaveland *et al*., 2003 [12]	Tanzania	Effect of vaccination	64	Free
Kaare *et al*., 2009 [27]	Tanzania	Assess vaccination coverage	80	Free
Kayali *et al*, 2003 [51]	Chad	Estimate the vaccination coverage	74	Free
Durr *et al*., 2009 [41]	Chad	Vaccination coverage	71	Free
Gsell *et al*., 2012 [24]	Tanzania	Demographic structure and vaccination coverage	78	Free

Table 3 provides the heterogeneity test results from the two groups of vaccination cost arrangement schemes. I² describes the percentage of variation across groups due to heterogeneity rather than chance. This study shows 99.9% heterogeneity between free and owner charged groups, indicating significant difference between the vaccination coverage in the two groups. Tau square is an estimate of the between-study variance in the meta-analysis. As Tau square between studied groups is larger than one (i.e. 1.45), it shows a substantial heterogeneity between the studied groups, while Tau squares within the free and charged groups were smaller than one (0.16 and 0.54, respectively).

**Table 3:** Statistical heterogeneity tests comparing vaccination coverage rates resulting from charged versus free-of-charge vaccination schemes.

Groups	No. studies	Vaccination coverage and 95% CI			Heterogeneity	
		Point estimate	Lower limit	Upper limit	I^2^	Tau^2^
Charged	8	0.18	0.17	0.19	98.45	0.48
Free	7	0.68	0.67	0.69	98.22	0.16
Overall	15	0.55	0.55	0.56	99.75	1.49

## Discussion

According to the World Health Organization (WHO), the adequate vaccination coverage of a dog population in a community vaccinated annually against rabies should be at least 70% in order to block the occurrence of an outbreak [1]. In this study dog accessibility for parenteral vaccination reflected by the ownership status and vaccination costs arrangement schemes were assessed to explore their influence on the realised vaccination coverage in Africa

When resources have to be allocated to the control of a disease, this should be done on scientific evidence. For instance, the organized efforts of the Pan American Health Organization (PAHO) in Latin America [28] and the Bohol Rabies Prevention and Elimination Project of the Philippines [29] have witnessed the possibility of reducing the incidence and burden of rabies with concerted efforts of experts. In Africa also, as growing scientific interest was shown through publications produced in the last few decades, it is possible to control rabies with organization of resources from different stakeholders together with a high local community involvement.

Accessibility of dogs is perceived to be the major operational constraint to achieve adequate coverage for dog vaccination against rabies through mass dog vaccination schemes[13]. Our study shows that the majority of dogs in Africa is free roaming but owned. Dogs having responsible owners are accessible for parenteral mass vaccination indicating the possibility of achieving the minimum proportion of dogs that ought to be vaccinated to reduce the incidence of rabies. However, it doesn’t mean that all owned dogs are presented for vaccination[30]. Oral rabies vaccination could be an option for those dogs that are difficult to capture, whether these dogs are owned or ownerless [31, 32]. As long as the proportion of ownerless dogs is less than 20% it is still possible to obtain sufficient immunity coverage by focussing on the mass vaccination of owned dogs. The relative impact of ownerless dogs could be studied by looking at the proportion of ownerless dogs compared to owned dogs in reported cases of human bites. For instance, in Nigeria only 9.7% of the dog bites could not be traced back to a dog with a responsible owner [33]. A study in South Africa showed that only a small proportion of dog bite reports resulted from unknown dogs [34]. In Chad, only 3% of the biting dogs were ownerless or from an unknown owner [35].

Studies referred in this review showed that the mean age of the African dog is between 1.9 and 3.4 years indicating an average turnover rate between 53% and 29%. These numbers are higher when compared to the turnover rates in industrialized countries as for instance, in North America and Europe where the dogs have an average life expectancy of respectively 4.5 years [36] and 5.7 years [37] resulting in an average turnover rate of 20%. Insight in the dog population demography, population size and turnover rates supports the selection of a vaccine in a vaccination scheme in terms of protection time period and frequency of required boosting to keep the required level of immunity.

Male dogs represent a considerable higher proportion of the African owned dog population than female dogs. Male dogs are more aggressive than female dogs and are, therefore, preferred for guarding and hunting. This might have implications in the transmission of rabies to humans and other dogs. For instance, a study in Chad showed that 80% of the human dog bites originated from male dogs [38]. Male dogs are also more likely to be diagnosed positive for rabies than females [39].The risk of acquiring an infection may possibly be influenced by males’ fighting over females during the breeding season [40].

According to the performed meta analysis, the vaccination costs recovery arrangement is one of the factors determining the proportion of dogs vaccinated against rabies in a community. In most African countries, where dog vaccination is not free of charge, the coverage is as low as 9% (Tanzania) [12]. The willingness of dog owners to pay for dog rabies vaccination is generally low as they perceive no direct economic and/or health benefit for themselves. This is supported by the knowledge that the majority of humans bitten were not bitten by their own dogs [29, 34].

The majority of the reviewed studies on charged vaccination schemes did not stated explicitly which costs were charged from the dog owners. In the study of Dürr et al. [41]in Chad, it was indicated that only 24% of the dogs was vaccinated during a parenteral mass dog vaccination campaign in which owners had to pay 21% of the vaccination costs themselves. Dog owners in Chad [42] were willing to pay ≈ 400–700 CFA francs per animal, while the average vaccination costs corresponded to 4000 CFA francs per animal (e.g. 10–17.5% of total costs). These findings also indicate the need for substantial subsidise to vaccinate the required >70% of dogs to interrupt rabies transmission.

Within the African context, the percentage of dogs vaccinated under free of charge vaccination schemes is much higher compared to the owner charged vaccination coverage. However, in developing countries in Asia, like Indonesia, a different situation was observed. Despite the application of a free of charge vaccination campaign [30] vaccination coverage remained as low as 33%. This difference might be explained by the diverse levels of awareness, beliefs and socio-economic factors among the different continents. Similarly, studies in Asia showed that educational level, dog ownership and veterinary service access are important factors affecting the vaccination coverage[29, 43].

The estimation of percentage of dogs vaccinated in owner charged schemes might have been slightly overestimated compared to the vaccination coverage in costs free schemes, because only owned dogs are considered in the case of owner charged vaccination schemes.

Despite the fact that puppies younger than 3 months of age are generally excluded from vaccination campaigns [41], they contribute to a significant proportion of the dog population. This will influence the vaccination coverage and also the risk of human rabies especially the risk for children who have more frequent contacts with puppies than adults. The general exclusion of this age group is often a result of acting upon vaccine manufactures’ guidelines and recommendations. Many rabies vaccines are licensed and approved for primary vaccination of dogs older than 3 months of age. However, it has been shown that most young puppies born from non-vaccinated mothers develop protective antibody titers after a vaccination as early as 4 weeks of age [44, 45].

A high vaccination coverage rate might not necessarily guarantee an effective rabies control. Studies have shown that African dogs experience reduced sero-conversions after rabies vaccination. The study of, for instance, indicated that only 71.9% of the vaccinated dogs in Nigeria developed neutralizing antibodies to rabies virus. The antibody titre depends on the time from vaccination, the technical efficacy of the vaccine used, the nutritional status, sex and age of the dogs [46, 47] also on the quality of the vaccine conservation. Furthermore, vaccination must be repeated more than once to effectively control rabies in a dog population.

A limitation of this study is the exclusion of unpublished reports and non-English articles in the review. Assessment of publications such as country reports of the Southern and Eastern Africa rabies group (SEARG) and the African Rabies Expert Bureau (AfroREB) could have provided additional information. However, as comparable reports are lacking or not accessible due to language barriers it could have created bias if only those country reports were included that were publicly accessible. Therefore, we focused our literature search on objective scientific articles that are internationally accessible.

The language selection criteria also excluded peer reviewed research papers published in French. This is could be a serious limitation of our study due to the fact that French is a very common language in African countries. A comparable systematic search in the French literature resulted in a selection of only 28 French papers (compared to the 1,239 hits resulted from the English search). Based on an evaluation of the English abstracts, only two publications [48, 49] fulfilled the remainder selection criteria as applied in this study. Main findings as presented in the abstracts of these papers were completely in line with the presented results.

## Conclusion

This review provided a comprehensive account on the dog rabies parenteral vaccination coverage, dog demography and ownership within the African situation. The main findings of this systematic review indicate that 1) there has been a growing scientific interest in dog rabies control in Africa during the last two decades, which reflects a positive development given the argument that scientific evidence dictates stakeholders in allocation of resources for control and prevention of infectious diseases, 2) only a small proportion of the African dog population is ownerless and, 3) puppies younger than 3 months of age constitute a considerable proportion of the African dog population, 4) male dogs are dominant in the African dog population and 5) the proportion of dogs vaccinated against dog rabies when vaccination is free of charge is closer to WHO recommendations compared to owner charged vaccination schemes. Therefore, as most dogs in Africa are owned and therefore accessible for parenteral vaccination, a high vaccination coverage can be obtained once the necessary financial arrangements are arranged through organized community participation and/or public funding arrangements.

## Supporting Information

S1 Checklist PRISMA Checklist. (DOC)
