# Supplementary material for: Review on Dog Rabies Vaccination Coverage in Africa: A Question of Dog Accessibility or Cost Recovery?
Source: PLoS Negl Trop Dis. 2015 Feb 3;9(2):e0003447. doi: 10.1371/journal.pntd.0003447 (PMC4315526; doi:10.1371/journal.pntd.0003447)
Supplement: S1 Checklist — (DOC) [file pntd.0003447.s001.doc]

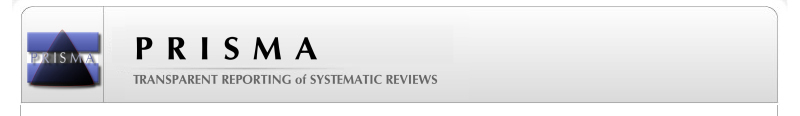
**Checklist S1: PRISMA Check list Flow Diagram for the Systematic review of ‘’Review on dog rabies vaccination coverage in Africa: A question of dog accessibility or cost recovery?’’**

**Screening**

**Included**

**Eligibility**

**Identification**

Records identified through database searching
(n = 9836 )

Additional records identified through other sources
(n = 0 )

Records after duplicates removed
(n =1239 )

Records screened
(n =19 )

Records excluded
(n = 1220 )

Full-text articles assessed for eligibility
(n =17 )

Full-text articles excluded, with reasons
(n =0 )

Studies included in qualitative synthesis
(n =16 )

Studies included in quantitative synthesis (meta-analysis)
(n = 11 )
